# Cerebrovascular Accidents After Orthotopic Liver Transplantation in Patients with Hepatopulmonary Syndrome: A Case Series

**DOI:** 10.3390/clinpract15010005

**Published:** 2024-12-29

**Authors:** Steffi K. Chan, Manuel M. Buitrago Blanco, Nicholas J. Feduska, Vatche G. Agopian, Samer S. Ebaid, Tisha Wang, Ami Tamhaney, Igor Barjaktarevic

**Affiliations:** 1 Department of Anesthesiology and Perioperative Medicine, David Geffen School of Medicine at UCLA, Los Angeles, CA 90095, USA; 2 Division of Neurocritical Care, David Geffen School of Medicine at UCLA, Los Angeles, CA 90095, USA; 3 Division of Liver and Pancreas Transplantation, Department of Surgery, David Geffen School of Medicine at UCLA, Los Angeles, CA 90095, USA; nfeduska@mednet.ucla.edu (N.J.F.);; 4 Division of Pulmonary and Critical Care Medicine, Department of Medicine, David Geffen School of Medicine at UCLA, Los Angeles, CA 90095, USA; tiwang@mednet.ucla.edu; 5 David Geffen School of Medicine at UCLA, Los Angeles, CA 90095, USA; atamhaney@mednet.ucla.edu

**Keywords:** hepatopulmonary syndrome, orthotopic liver transplant, end-stage liver disease, cerebrovascular accident, stroke

## Abstract

**Background:** Hepatopulmonary syndrome (HPS), defined by the presence of pulmonary vascular dilatations that cause right-to-left transpulmonary shunting of venous blood with a consequential increase in the alveolar–arterial oxygen gradient, is a relatively frequent complication of chronic liver disease. While orthotopic liver transplantation (OLT) is indicated and often curative in HPS patients with end-stage liver disease (ESLD), little is known about the peri- and post-operative-period risks of CVA in OLT recipients with HPS. **Case Presentation**: We report a case series of five non-consecutive OLT recipients with HPS who developed ischemic and/or hemorrhagic CVAs during or shortly after OLT, raising concern that the risks of neurological complications remain increased even after OLT. **Conclusions:** Our case series hopes to highlight the importance of close vigilance in this subset of patients, at a time when there may be multiple issues to be addressed in facilitating post-operative recovery.

## 1. Introduction

Hepatopulmonary syndrome (HPS), defined by the triad of chronic liver disease, pulmonary vascular dilatation, and increased alveolar–arterial oxygen gradient, is frequently observed among cirrhotic patients awaiting liver transplantation with an estimated prevalence of between 4–19% [1]. Intrapulmonary vascular dilatations and abnormal angiogenesis [2] lead to ventilation–perfusion (V/Q) mismatch, diffusion limitation, and anatomic shunting. HPS portends a poor prognosis, with a mortality rate more than twice as high as patients with a similar severity of cirrhosis [3,4]. Orthotopic liver transplantation (OLT) is the only definitive cure to prevent further progression of the disease and is advocated before the development of severe HPS. Severe hypoxemia due to HPS (PaO_2_ < 60 mmHg) is considered an indication for OLT with model for end-stage liver disease (MELD) exception points granted to expedite transplantation [3,4]. However, resolution of HPS post-OLT is not immediate and may take several months [5]. Increased risks of cerebrovascular accidents (CVAs) in end-stage liver disease (ESLD) patients have been reported; however, little is known about the risk of neurological complications in early post-OLT period among patients with HPS.

We present a case series of five patients from 2007 to 2022 with known HPS who developed CVA in the immediate or early post-operative period. Our case series hopes to highlight the importance of close vigilance in this subset of patients, at a time when there may be multiple issues to be addressed in facilitating post-operative recovery. These cases have been presented in accordance with the CARE checklist (Supplementary Materials).

## 2. Case Series

### 2.1. Case 1

A 61-year-old man with ESLD due to cirrhosis secondary to hepatitis C was diagnosed with severe HPS and chronic respiratory failure. Transthoracic echocardiogram (TTE) indicated transpulmonary shunting, and a 100% oxygen shunt study indicated a shunt fraction of 23%. He had a baseline PaO_2_ of 55 mg on room air, but on admission, he required a fraction of inspired oxygen (FiO_2_) of 0.45 via a high-flow nasal cannula (HFNC), achieving a SpO_2_ of 82–94%. He had no previous CVA nor risk factors for CVA such as diabetes mellitus (DM), hypertension, hyperlipidemia, or atrial fibrillation.

**Figure 1:**
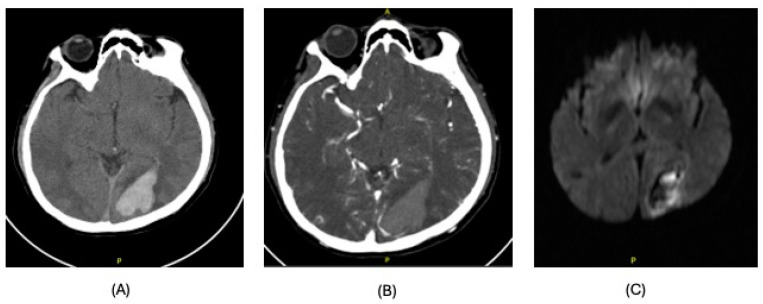
Imaging findings, case 1. Left occipital intraparenchymal hematoma. (**A**): CT of brain; (**B**): CT angiography; (**C**): MRI of brain.

The patient was admitted for OLT with a physiologic MELD score of 12. After an uneventful OLT, the patient required prolonged mechanical ventilation and was extubated on post-operative day (POD) 22. He was discharged to subacute rehabilitation on POD 34. On POD 39, he was readmitted for a left headache with right visual field defects and hemianopia. Computer tomography (CT) of the brain revealed a left occipital intraparenchymal bleed (Figure 1). His coagulation profile was normal at the time of the event. He was treated conservatively and discharged 2 months after OLT with persistent right hemianopia.

### 2.2. Case 2

A 33-year-old woman with ESLD secondary to metabolic-dysfunction-associated steatohepatitis (MASH), a history of gastro-esophageal reflux disease (GERD), and chronic kidney disease (CKD) was diagnosed with moderate HPS (PaO_2_ 76 mmHg on room air) based on a TTE with agitated saline (bubble) study, which showed transpulmonary shunting. Bubble filters had been placed on all intravenous cannulas in view of her history of HPS.

**Figure 2:**
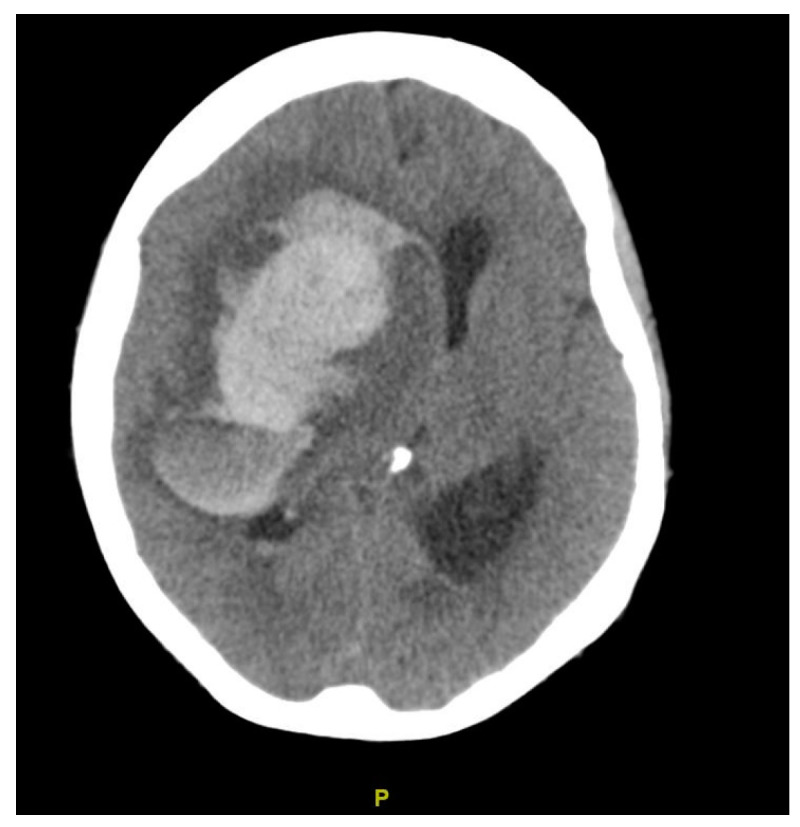
CT of brain, case 2. Large right frontal hematoma with severe surrounding mass effect, leftward shift of the midline and right uncal herniation.

She was initially admitted for an unreduced rectal prolapse and gastrointestinal bleeding. Prior to OLT, she developed fluid overload from acute kidney injury and required intubation and continuous renal replacement therapy 4 days prior to surgery. She underwent OLT on her 16th day of admission, with a physiologic MELD score of 30. Although the surgery was uneventful, she was persistently drowsy post-operatively and then developed a dilated right pupil on POD 3. CT of the brain revealed a large right frontal hematoma with a severe surrounding mass effect, a leftward shift of the midline, and right uncal herniation (Figure 2). She was thrombocytopenic (platelets = 44 × 10^9^/L), although the rest of her coagulation profile and graft function was satisfactory. A neurosurgical consultation was sought. In view of her poor prognosis even with surgery, comfort care measures were advised, and she passed away 1 day later.

### 2.3. Case 3

A 31-year-old woman with ESLD secondary to biliary atresia, status post-kasai procedure at 3 months of age, complicated by recurrent cholangitis and decompensated cirrhosis, and a history of GERD was diagnosed pre-operatively with HPS. A TTE bubble study showed significant shunting, and arterial blood gas revealed a PaO_2_ of 64 mmHg on room air. Technetium ^99m^Tc albumin aggregated (^99m^Tc-MAA) scintigraphy showed a right-to-left shunt of 15.6%. She was treated with oral garlic tablets for moderate HPS.

**Figure 3:**
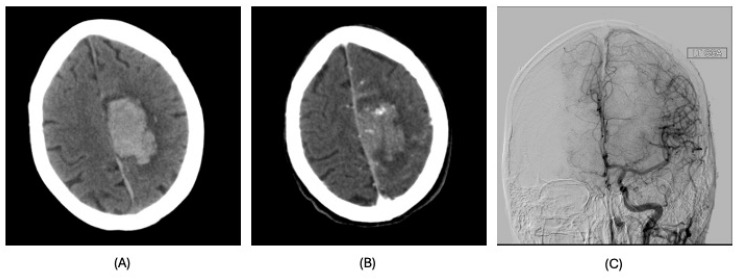
Imaging findings, case 3. Left frontoparietal parenchymal hematoma with locoregional mass effect and mild rightward midline shift. Multiple serpiginous vessels in the region of the hematoma; no arteriovenous malformations. (**A**): CT of brain; (**B**): CT angiography; (**C**): digital subtraction angiography.

She presented from home for OLT with a physiologic MELD of 33. Bubble filters were applied to intravenous lines during her admission. After an uneventful transplantation, she developed a headache on POD 5, followed by right hemiparesis the next day. Physical examination revealed motor deficits in her right upper and lower extremities, strength 0–1 out of 5, and decreased sensation to pinprick on her right lower extremity. CT of the brain revealed a left frontoparietal parenchymal hematoma with locoregional mass effect and mild rightward midline shift (Figure 3). Although multiple serpiginous vessels were seen in the region of the hematoma, vascular malformations were not identified on cerebral angiogram. Her coagulation profile was normal. She underwent an endoscopic left craniotomy for evacuation of hematoma and was prescribed levetiracetam for seizure prophylaxis. She required rehabilitation for 7 months and was discharged with residual right-sided weakness.

### 2.4. Case 4

A 62-year-old man with ESLD secondary to MASH complicated by hepatocellular carcinoma (HCC), history of CKD, and asthma was admitted for OLT. While no significant alveolar–arterial (A-a) gradient was found on pre-operative arterial blood gas analysis, mild HPS was diagnosed on a pre-operative TTE bubble study, showing a pulmonary shunt. Prior to his OLT, he had been admitted after an unwitnessed fall and was found to have a small traumatic subarachnoid hemorrhage upon CT scanning. He was monitored closely in the intensive care unit (ICU) and discharged home after 2 weeks with no neurological deficits. He underwent a relatively uneventful OLT 6 weeks after his fall. At the time of surgery, his calculated MELD was 31.

**Figure 4:**
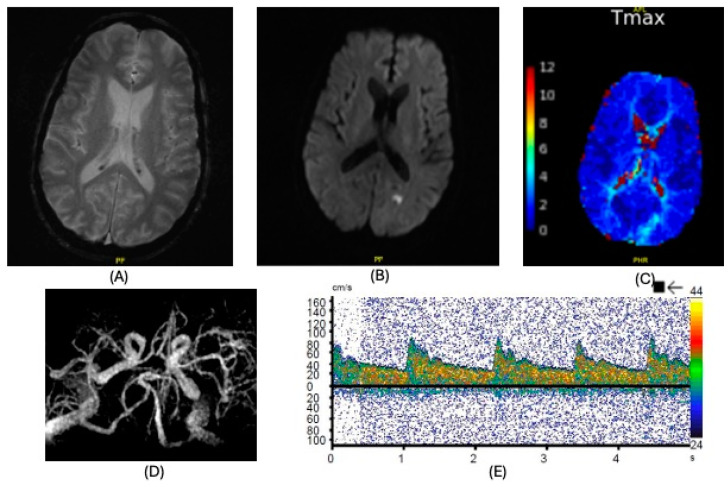
MRI of brain, case 4. Small infarct in the left occipital lobe. No significant stenoses of occlusions. (**A**): MRI diffusion-weighted imaging; (**B**): MRI gradient echo; (**C**): MRI time-to-maximum; (**D**): MR angiography; (**E**): transcranial Doppler.

Subsequently, on POD 10, he was noted to be aphasic with word-finding difficulty. Upon magnetic resonance imaging (MRI), there was no evidence of an intracranial bleed, but a small infarct was seen in the left occipital lobe (Figure 4A–C). Magnetic resonance (MR) angiography excluded significant stenoses or occlusions in his brain and neck vessels (Figure 4D). However, transcranial Doppler revealed two spontaneous emboli in his right middle cerebral artery (MCA) (Figure 4E). Following the injection of 10cc of agitated saline into the antecubital vein, more than 100 high-intensity transient signals (HITS) were also seen in both MCAs, indicative of cerebral air emboli. Further advice from neurology was sought, and his deficits were attributed to a cardioembolic stroke of a posterior cerebral artery branch. Apart from thrombocytopenia (platelets = 85 × 10^9^/L), his coagulation profile was unremarkable at the time of the event. The patient received 1 month of in-house rehabilitation. At discharge, he was mildly dysphoric but fluent with no other deficits.

### 2.5. Case 5

A 49-year-old man with ESLD secondary to alcoholic cirrhosis had a history of a transient ischemic attack and glaucoma. A TTE bubble study revealed a large degree of late right-to-left shunting, suggestive of a transpulmonary shunt with concomitant small patent foramen ovale (PFO). He was initially admitted for abdominal pain from a rectus sheath hematoma and intubated for acute-on-chronic respiratory failure. He underwent an uneventful OLT 8 days after admission with a calculated MELD score of 26 and was monitored in ICU post-operatively. He was extubated on POD 3 to HFNC. Bubble filters were used for the administration of intravenous medication.

**Figure 5:**
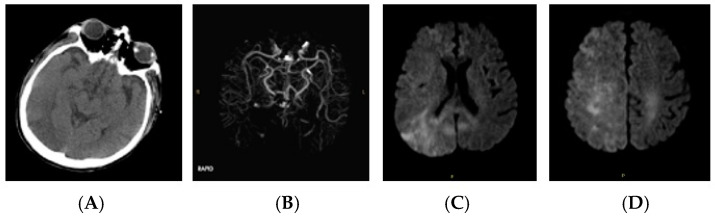
Imaging findings, case 5. Evolving subacute infarcts throughout the right cerebral hemisphere. No significant stenosis, occlusion, or aneurysms. (**A**): CT of the brain; (**B**): CT angiography; (**C**,**D**): MRI of the brain, diffusion-weighted imaging.

On POD 9, shortly after central venous catheter removal, he developed altered mental status, a right gaze preference, and left-sided flaccid paralysis (strength 0–1/5). CT of the brain showed evolving subacute infarcts throughout the right cerebral hemisphere (Figure 5). No significant stenosis, occlusion, or aneurysms were noted on CT angiography of the neck and brain. He was thrombocytopenic (platelets = 34 × 10^9^/L) with an elevated activated partial thromboplastin time (APTT) of 40.7 s. The rest of his coagulation profile was normal. More than 300 microemboli in the cerebral circulation were noted during transcranial Doppler, and a concurrent bubble study suggested that a large right-to-left shunt was present. An electroencephalogram (EEG) showed severe attenuation over the right hemisphere and excluded epileptiform activity. Due to his neurological status, he required reintubation and underwent tracheostomy 3 days after his stroke diagnosis. He was prescribed aspirin, atorvastatin, and levetiracetam. He underwent extensive in-house physical rehabilitation for 2 months. He was decannulated 1 month after his CVA, and at discharge on POD 84, he was able to ambulate with a front-wheeled walker and moderate assistance.

## 3. Discussion

HPS is characterized by pulmonary vasodilation and subsequent hypoxemia in the setting of hepatic dysfunction. It is characterized by the triad of hypoxemia with an abnormal alveolar–arterial (A-a) gradient, the presence of intrapulmonary shunting, and chronic liver disease [5,6]. Although this syndrome is not completely understood, the pathophysiology is characterized by pulmonary microvascular dilatation and angiogenesis, which lead to an intrapulmonary right–left shunt predominantly affecting the lower lung zones. The frequency of this disorder among patients with cirrhosis varies from 4% to 47% and may be overall underdiagnosed due to other comorbidities and pulmonary complications of liver disease that mask its presence [4].

Neurologic complications of HPS have been described, although mostly at the level of case reports with an overall paucity of the literature. While brain abscesses may occur [7], the risk of ischemic strokes, due to paradoxical embolisms and intracerebral hemorrhages, due to a combination of coagulopathy and possibly the proliferation of small perforating arteries and diffuse arteriovenous (AV) shunting without AV malformation [8], may be increased in HPS [9,10]. A previous meta-analysis on cirrhosis and stroke found that liver disease is an independent risk factor [11], predisposing patients to CVAs by an additional 24% compared to healthy controls, possibly a consequence of coagulopathy and thrombocytopenia characterizing end-stage liver disease. Nevertheless, most of these complications arise before liver transplantation, and, while HPS patients are prone to significant peri-operative morbidity and mortality [11,12], HPS generally improves over time, and the risk of complications decreases over time with the recovery of liver function. Theoretically, correction of clotting factor production and recovery of platelet numbers, which occurs shortly after successful OLT, would lessen the overall bleed risk.

Overall, cerebrovascular complications are rare and have been reported in 1.8–4% of OLT recipients [13]. Reviewing the outcome data of 943 consecutive OLT recipients at UCLA in the period between 2007–2011, post-operative strokes in the extended period of two years after OLT were documented in 4% of cases (unpublished data). Higher risk of these events has been reported in older recipients and with pretransplant diabetes, similar to the general population [14]. Ischemic stroke is less common than intracranial hemorrhage. This difference in incidence may be related to the increased incidence of intracranial bleeding risk factors, such as thrombocytopenia [15], coagulopathy, and sepsis [16].

The risk of post-OLT CVAs, specifically in patients with HPS, has not been previously established. Here, we report on a case series of cerebrovascular accidents in the peri-operative period in OLT recipients with known HPS (Table 1) and discuss several interesting observations potentially relevant to the management of HPS patients undergoing OLT.

First, these patients with HPS had CVAs shortly after OLT, which may suggest a potentially increased prevalence and higher risk of post-operative CVA in HPS compared to OLT recipients without HPS. While the actual incidence of CVA in OLT recipients with HPS in this retrospective database analysis cannot be accurately assessed, of 85 non-consecutive ESLD patients diagnosed with HPS who received OLT at our center between 2007–2022, we identified 5 patients with post-operative CVA within 1 year of OLT (estimated prevalence of 5.9%), which may be relatively higher in comparison to the reported prevalence of CVA in all liver transplant recipients [13]. In our analysis of 943 consecutive OLTs from 2007 to 2011, 3 out of 29 patients (10.3%) with HPS developed post-operative CVAs in the two years after OLT, resulting in an odds ratio of 3.08 of CVA compared to the non-HPS transplant population (unpublished data). Although this data should be interpreted cautiously, evidence on the incidence of post-operative CVAs in this subgroup of patients is limited and is a potential area for exploration.

Although HPS generally resolves over time post-OLT, the shunt and HPS pathophysiology persists for weeks to months, and patients may remain at risk of complications specific to HPS. In this case series, the earliest reported case occurred on POD 3, while another patient presented more than a month after surgery, with a mean ± standard deviation (SD) time post-OLT of 13.2 ± 14.7 days. Four out of five cases presented within 10 days of OLT with a variety of symptoms. Although it is difficult to determine the period during which HPS patients may be at increased risk of CVA post OLT—not only because of the small patient cohort but also due to the highly variable time needed for the resolution of HPS—one could speculate that HPS patients may be at risk of neurologic complications for weeks after OLT.

Secondly, all events were unexpected and clinically significant. All five patients suffered a clinically significant stroke, and four patients (80%) had long-term sequelae, including one death (20%). Interestingly, there was a wide range of physiological MELD scores at transplant (13 to 38), although the degree of shunting did not seem to correlate directly with MELD severity. The death outcome was in the patient with the highest MELD score (physiologic MELD score: 38), who had mild HPS.

Thirdly, all patients did not have significant risk factors for CVA. None of the five patients had traditional risk factors for stroke such as diabetes, coronary artery disease, hypertension, hypercholesterolemia, obesity, or a history of tobacco use. Similarly, none of these patients had significant post-operative coagulopathy, although 60% of them had thrombocytopenia (platelets < 150 × 10^3^/mL). Two patients had a history of CVA, and these were considered clinically not relevant to preclude the subsequent OLT; one of these was the consequence of a subarachnoid hemorrhage prior to OLT, while the post-OLT CVA was an ischemic event. The other patient with pre-operative CVA had a history of transient ischemic attack (TIA) initially, followed by a more significant embolic stroke in the post-OLT period. Certainly, the TIA history could also be related to the underlying HPS, but these observations bring up the possibility that pre-operative CVA in this population of HPS patients with ESLD may be associated with an increased post-operative risk of CVA.

Of note is the fact that only the first patient had HPS severe enough to qualify for MELD exception points. Three other patients (60%) had calculated MELD scores of >30 and were prioritized for liver transplants for reasons other than HPS. Interestingly, similar cases of mild HPS developing cerebral embolic events post-OLT have been reported [1].

Mild HPS, defined as having a PaO_2_ of ≥80 mmHg or an alveolar–arteriolar gradient of ≥15 mmHg on ambient air, may not be detected clinically [17]. Previous studies have shown that in patients with biopsy-proven cirrhosis, the incidence of a positive contrast echocardiogram may be as high as 34%, of which nearly 40% may have PaO_2_ ≥ 80 mmHg [1]. In these patients with “subclinical” HPS, only 54% had other features of abnormal vascular dilatations such as spider naevi. Dyspnea is also nonspecific and common in patients with advanced liver disease. The identification of HPS requires a contrast-enhanced transthoracic echocardiography, which is not routinely performed in all patients being evaluated for OLT. Thus, patients with mild HPS could easily be missed. Given the significant likelihood of a positive contrast echocardiogram, even in patients who have minimal symptoms, a more extensive pre-operative workup involving the routine use of contrast echocardiography may be beneficial in the general liver transplant population, as even patients with subclinical HPS may be at risk of post-operative CVA.

Finally, with this small case series, we report that the CVA in the HPS population can be a consequence of both embolic as well as a hemorrhagic etiology. Three (60%) patients were diagnosed with intracranial hemorrhage (ICH), while emboli leading to cerebral infarcts were present in the remaining patients. Findings from previous studies have highlighted several mechanisms that may place HPS patients at risk of both ischemic and hemorrhagic stroke.

Abnormally dilated pulmonary vessels are less effective in filtering emboli from the circulation. The increase in the diameter of capillary vessels has been found to reach up to 100 um in diameter [18], more than 10 times that of the normal lung vascular capillary vessels. The loss of this filter to the systemic circulation puts the patient at risk of air emboli or clots entering the cerebral vasculature. Similarly, if a clinically significant number of gas bubbles were to lodge in capillaries and impede oxygen delivery to delicate neurons, an ischemic stroke could develop. This mechanism is similar to that in pulmonary arteriovenous malformations (PAVMs), which have been identified as a rare cause of ischemic strokes [19,20]. It is also plausible that the hemorrhagic events may reflect secondary bleeding in areas of ischemia caused by the embolic phenomena.

Several studies have identified intracranial shunting in patients with HPS, both focal and diffuse [8]. Similar to the lungs, the widespread proliferation of small cerebral arteries and diffuse arteriovenous shunting have been found on contrast imaging [8,21]. Cerebral vasculature may dilate, become tortuous, produce shunt physiology, and often produce arteriovenous malformations [22]. Abnormal cerebral vessels may also be more friable. These changes have been hypothesized to contribute to cerebral ischemia due to V/Q mismatch. Coupled with profound coagulopathy, thrombocytopenia, fluid overload, a loss of normal cerebral autoregulation, and major hemodynamic swings in the peri-operative period, these factors can all lead to a significant risk of hemorrhagic stroke and intracranial bleeds [18,19,20]. Interestingly, even though the average age of these patients was 47.2 years, all cases of ICH had bleeds in the lobar region, which is more likely to be associated with cerebral amyloid angiopathies, microbleeds, and microinfarcts in the elderly population. Of note is the fact that none of the patients diagnosed with ICH (cases 1, 2, and 3) had an abnormal coagulation profile on the day of diagnosis. This may be suggestive of an underlying structural predisposition to ICH in these patients. [22]

Given the myriad ways in which HPS and stroke can present, it is impossible to commit exclusively to a single mechanism, and a multifactorial process is likely. Since the first case of recurrent ICH in the setting of HPS was reported more than 30 years ago [9], there have been isolated case reports, with varying stroke patterns—ranging from multiple recurrent intracerebral hemorrhages [23] to diffuse mixed microhemorrhages and acute ischemic lesions [8]. However, it is fair to say that these patients are at risk of both ischemic and hemorrhagic stroke and should be closely monitored post-operatively. The use of bubble filters, careful administration of intravenous medication, and due diligence during high-risk procedures may help to prevent systemic emboli, while careful management of hemodynamics, coagulopathy, and avoiding thrombocytopenia may decrease the chance of intracranial bleeding.

## 4. Limitations

Although our findings expand the currently available literature about the risks of cerebrovascular accidents in HPS patients who have undergone liver transplant, this small case series of non-consecutive OLT recipients does not allow us to draw any definitive conclusions with regards to the risk, mechanism, or true prevalence of this disorder. We also were not able to obtain a histopathologic analysis of brain tissue, which could provide better insight into the mechanism of each stroke.

## 5. Conclusions

Although CVA after OLT appears to be rare, it can be functionally devastating and, based on the reported case series, it may happen more frequently in the early post-operative period in liver transplant recipients with HPS and can occur unexpectedly with serious consequences. Management is multifaceted, involving the prevention of systemic embolic events and tight physiological control, as well as close neurological monitoring. More data are required to better assess the risks and mechanisms of CVA after OLT in HPS.

## Figures and Tables

**Table 1:** Summary of patients.

Case no.		1	2	3	4	5
Biodata	Age (years), Gender at Birth	45 Female	33 Female	31 Female	60 Male	49 Male
	Etiology of End- Stage Liver Disease (ESLD)	Hepatitis C	Metabolic Dysfunction-associated Steatohepatitis (MASH)	Biliary Atresia	MASH complicated by Hepatocellular Carcinoma	Alcohol-associated Cirrhosis
	Medical History Risk factors (1)	none	Chronic Kidney Disease	none	Previous post- traumatic small subarachnoid hemorrhage 6 weeks prior to OLT	Previous transient ischemic attack
Hepatopulmonary Syndrome (HPS) History	Oxygen Requirement at index admission	FiO_2_ 0.45 via High Flow Nasal Cannula, SpO2 82–94%	PaO_2_ 76 mmHg on room air, SpO2 93%	PaO_2_ 66 mmHg on room air	PaO_2_ 88 mmHg on room air	PaO_2_ 66 mmHg on room air
	Diagnostic Findings	Transthoracic Echocardiography (TTE): Patent Foramen Ovale (PFO) + HPS Shunt study: 23.2% shunt fraction	TTE bubble study: Mild right-to-left shunting at rest, consistent with transpulmonary shunt	TTE: PFO + HPS, large shunt 99mTc-MAA scintigraphy: right-to-left shunt, 15.6%	T TE bubble study: positive with late transition, consistent with transpulmonary shunt	TTE bubble study: large degree of right-to-left shunting, likely due to both interatrial and transpulmonary shunt
	Management	Bubble filters	Bubble filters	PO garlic 1000 mg two times a day	none	PO garlic 1000 mg two times a day
Orthotopic Liver Transplant (OLT) History	Physiologic MELD score at transplant	12	30	33	31	26
	Complications of ESLD	Hepatorenal Syndrome	Ascites, Esophageal Varices	Ascites, esophageal varices, hyponatremia, thrombocytopenia, recurrent cholangitis	Hepatic encephalopathy, esophageal and gastric varices, thrombocytopenia	Ascites, esophageal varices, pancytopenia
	Mechanical Ventilation days	23	N/A	1	5	7
	ICU stay Post-OLT (days)	27	4	12	17	35
Cerebrovascular Accident (CVA) History	Date of CVA (POD)	39	3	5	10	9
	Patient Location	Subacute rehabilitation unit	Inpatient, ICU	Inpatient, ICU	Inpatient, medical floor	Inpatient, ICU
	Coagulation Profile	INR: 1.1 Plt: 189 × 109/L	INR: 1.2 Plt: 44	INR: 1.3 Plt: 156	INR: 1.1 Plt: 95	INR: 1.1 APTT: 40.7 s Plt: 34
	Liver Function Test	AST: 40 U/L ALT: 115 U/L ALP: 301 U/L Bilirubin (total): 0.9 mg/dL	AST: 93 ALT: 134 ALP: 71 Bilirubin (total): 9.0	AST: 13 ALT: 93 ALP: 69 Bilirubin (total): 3.3	AST: 46 ALT: 59 ALP: 210 Bilirubin (total): 2.1	AST: 14 ALT: 60 ALP: 79 Bilirubin (total): 2.0
	Diagnosis	Lobar intracranial hemorrhage (ICH)	Basal Ganglia ICH	Lobar ICH	Embolic ischemic stroke (IS)	Embolic IS
	Clinical Syndrome	Left headache, right hemianopia	Persistent drowsiness post-OLT, GCS E1VTM2, dilated right pupil	Headache, right hemiparesis, right lower limb sensory deficits	Aphasia	Altered mental status, right gaze preference, left flaccid paralysis
	Imaging	CT scan	CT scan	CT scan MRI CT angiography	CT scan MRI MR angiography	CT scan CT angiography MRI
	Findings	CT: Left occipital intraparenchymal hematoma Volume: 15 mL	CT: Large right frontal temporal parenchymal hematoma, severe surrounding mass effect with leftward midline shift, right uncal herniation Volume: 93.5 mL	CT: Right frontoparietal parenchymal hematoma with locoregional mass effect, rightward midline shift Volume: 25 mL	CT: Left occipital infarct Transcranial doppler: 2 spontaneous emboli, more than 100 high intensity transient signals (HITS) in bilateral middle cerebral arteries (MCAs) after agitated saline	CT: Right parietal lobe hypodensity Transcranial doppler: more than 300 HITS in bilateral MCAs after agitated saline
	Management	Conservative	Non-operative in view of poor prognosis	Endoscopic left craniotomy for evacuation of hematoma	Conservative	Conservative management; tracheostomized and decannulated 1 month after CVA
Post-CVA Status	Length-of-Stay (LOS)	Hospital LOS 28 days	Post-OLT LOS 4 days	Post-OLT LOS 22 days	Post-OLT LOS 45 days	Post-OLT LOS 84 days
	Sequelae, Discharge	Discharged with persistent right hemianopia	Death discharge	Discharged with residual right-sided weakness	Discharged with mild dysphoria, no other deficits	Discharged, able to ambulate with a walker and moderate assistance
	Status at 360 Days	Alive	Deceased	Alive	Alive	Alive at 4 months

(1) Diabetes mellitus, coronary artery disease, hypertension, peripheral vascular disease, hyperlipidemia, chronic kidney disease, atrial fibrillation, tobacco use. MELD: model for end-stage liver disease; ICU: intensive care unit; FiO_2_: fraction of inspired oxygen; SpO2: oxygen saturation; INR: international normalized ratio; AST: aspartate aminotransferase; ALT: alanine aminotransferase; ALP: alkaline phosphate; CT: computed tomography; MRI: magnetic resonance imaging.

## Data Availability

The underlying data were abstracted from the electronic medical record at Ronald Reagan UCLA Medical Center. All data generated or analyzed during this study are included in this article. Further enquiries can be directed to the corresponding author.

## Supplementary Materials

The following supporting information can be downloaded at: https://www.mdpi.com/article/10.3390/clinpract15010005/s1, CARE checklist of information to include when writing a case report.

## List of Abbreviations

HPS	Hepatopulmonary syndrome
V/Q	Ventilation–perfusion
OLT	Orthotopic liver transplantation
PaO_2_	Partial pressure of oxygen
MELD	Model for end-stage liver disease
CVA	Cerebrovascular accident
ESLD	End-stage liver disease
TTE	Transthoracic echocardiography
FiO_2_	Fraction of inspired oxygen
HFNC	High-flow nasal cannula
DM	Diabetes mellitus
POD	Post-operative day
CT	Computer tomography
MASH	Metabolic-dysfunction-associated steatohepatitis
GERD	Gastroesophageal reflux disease
CKD	Chronic kidney disease
99mTc-MAA	Technetium 99mTc albumin aggregated
HCC	Hepatocellular carcinoma
ICU	Intensive care unit
MRI	Magnetic resonance imaging
MR	Magnetic resonance
MCA	Middle cerebral artery
HITS	High-intensity transient signals
PFO	Patent foramen ovale
APTT	Activated partial thromboplastin time
EEG	Electroencephalogram
A-a	Alveolar–arterial
AV	Arteriovenous
SD	Standard deviations
TIA	Transient ischemic attack
ICH	Intracranial hemorrhage
PAVM	Pulmonary venous malformations
