# Supplementary material for: Cerebrovascular Accidents After Orthotopic Liver Transplantation in Patients with Hepatopulmonary Syndrome: A Case Series
Source: Clin Pract. 2024 Dec 29;15(1):5. doi: 10.3390/clinpract15010005 (PMC11764223; doi:10.3390/clinpract15010005)
Supplement: Supplementary file 1 [file clinpract-15-00005-s001.zip › clinpract-3249379-supplementary.pdf]

CARE Checklist of information to include when writing a case report

CARE case report guidelines

(cc) BY-NC-ND

Topic	Item	Checklist item description	Reported on Line
Title	1	The diagnosis or intervention of primary focus followed by the words "case report"	1-4
Key Words	2	2 to 5 key words that identify diagnoses or interventions in this case report, including "case report"	33-34
Abstract (no references)	3a	Introduction: What is unique about this case and what does it add to the scientific literature?	19-25
	3b	Main symptoms and/or important clinical findings	27
	3c	The main diagnoses, therapeutic interventions, and outcomes	27-28
	3d	Conclusion—What is the main "take-away" lesson(s) from this case?	30-32
Introduction	4	One or two paragraphs summarizing why this case is unique (**may include references**)	37-120
Patient Information	5a	De-identified patient specific information.	123, 142, 185, 212, 247
	5b	Primary concerns and symptoms of the patient.	133, 174, 193-195, 221, 255-256
	5c	Medical, family, and psycho-social history including relevant genetic information	123, 143, 186-187, 213, 248
	5d	Relevant past interventions with outcomes	127, 171-172, 190-192, 216-217, 250-251
Clinical Findings	6	Describe significant physical examination (PE) and important clinical findings.	133, 174, 193-195, 221, 255-256
Timeline	7	Historical and current information from this episode of care organized as a timeline	N/A
Diagnostic Assessment	8a	Diagnostic testing (such as PE, laboratory testing, imaging, surveys).	134, 175-177, 196-197, 222-228, 256-264
	8b	Diagnostic challenges (such as access to testing, financial, or cultural)	N/A
	8c	Diagnosis (including other diagnoses considered)	134-135, 175-177, 196-197, 223, 257
	8d	Prognosis (such as staging in oncology) where applicable	N/A
Therapeutic Intervention	9a	Types of therapeutic intervention (such as pharmacologic, surgical, preventive, self-care)	136, 179, 199-201, 231-232, 264-267
	9b	Administration of therapeutic intervention (such as dosage, strength, duration)	136, 179, 199-201, 231-232, 264-267
	9c	Changes in therapeutic intervention (with rationale)	N/A
Follow-up and Outcomes	10a	Clinician and patient-assessed outcomes (if available)	136-137, 180, 202, 232-233, 268-269
	10b	Important follow-up diagnostic and other test results	N/A
	10c	Intervention adherence and tolerability (How was this assessed?)	N/A
	10d	Adverse and unanticipated events	N/A
Discussion	11a	A scientific discussion of the strengths AND limitations associated with this case report	323-326, 447-452
	11b	Discussion of the relevant medical literature **with references**.	292-322
	11c	The scientific rationale for any conclusions (including assessment of possible causes)	336-445
	11d	The primary "take-away" lessons of this case report (without references) in a one paragraph conclusion	454-460
Patient Perspective	12	The patient should share their perspective in one to two paragraphs on the treatment(s) they received	N/A
Informed Consent	13	Did the patient give informed consent? Please provide if requested	Yes ☐ No ☒
